# Compound and Dose-Dependent Effects of Two Neonicotinoid Pesticides on Honey Bee (*Apis mellifera*) Metabolic Physiology

**DOI:** 10.3390/insects10010018

**Published:** 2019-01-08

**Authors:** Steven C. Cook

**Affiliations:** USDA-ARS, Bee Research Laboratory, 10300 Baltimore Avenue, Beltsville, MD 20705, USA; steven.cook@ars.usda.gov

**Keywords:** carbon dioxide, glucose, glycogen, lipids, neurophysiology, protein, respiration

## Abstract

Use of neonicotinoid pesticides is now ubiquitous, and consequently non-targeted arthropods are exposed to their residues at sub-lethal doses. Exposure to these neurotoxins may be a major contributor to poor honey bee colony health. Few studies have explored how sub lethal exposure to neonicotinoids affects honey bee metabolic physiology, including nutritional and energetic homeostasis, both of which are important for maintaining colony health. Reported here are results from a study of chronic oral exposure of honey bees to two sub lethal concentrations of clothianidin and imidacloprid. Neonicotinoids altered important aspects of honey bee nutritional and metabolic physiology in a compound and dose-dependent manner; both compounds at low doses reduced honey bee body weight. Low-dose clothianidin exposure resulted in bees having protein, lipids, carbohydrates, and glycogen levels similar to newly emerged bees. High-dose clothianidin exposure lowered lipids and glycogen content of bees. High-dose imidacloprid exposure resulted in bees having depressed metabolic rate. Low-dose imidacloprid exposure resulted in bees consuming low and high levels of protein and carbohydrate rich foods, respectively. Results suggest neonicotinoids interfere with honey bee endocrine neurophysiological pathways. Compound and dose-dependent effects might represent respective chemical structural differences determining an observed effect, and thresholds of compound effects on honey bee physiology.

## 1. Introduction

Neonicotinoids are a nascent class of neurotoxic pesticides introduced in the 1990s for agricultural use against a range of insect pest species [1]. Since then, their use has become widespread in both agricultural and domestic settings [1,2]. Neonicotinoids act as agonists of insect nicotinic acetylcholine receptors (nAChRs), binding irreversibly to receptors and causing uncontrollable muscle activity, paralysis, and eventual death [3,4]. Although neonicotinoid pesticides were designed to target nAChRs of pest species such as aphids, “leakage” in pesticide site selectivity across taxa, and diverse actions of neonicotinoids on insect nAChRs, raised concerns of possible negative effects of neonicotinoids on beneficial species [5]. Additionally, neonicotinoid-based pesticides are systemic, imparting their presence throughout tissues of treated plants, including pollen, nectar, and other plant secretions collected by pollinators, such as honey bees. Residues of systemic pesticides can be present at a range of sub lethal concentrations in these types of resources [6,7,8,9,10].

Because of potential exposure of “non-targets” to neonicotinoids, much research has been undertaken to discern the effects to honey bees from sub lethal exposure to neonicotinoids in both laboratory, and semi- and full-field level studies [6,11,12,13,14]. Sub-lethal exposure to neonicotinoids can cause neurological disorders in honey bees [15,16], aligned with their cholinergic mode of action on the central nervous system (CNS). Depressed cognitive abilities, including learning, memory and habituation, olfaction and gustation, and navigation and orientation, can alter foraging behaviors [17,18] (e.g., precocious foraging), which may have detrimental consequences to colony endurance [19].

Sublethal neonicotinoid exposure can also affect honey bee metabolic physiology [15]. Larvae transiently exposed to a low dose of imidacloprid showed down-regulation of genes modulating rates of glycolysis, lipid metabolism, and protein synthesis [20]. Adults exposed to clothianidin and imidacloprid showed altered expression of metabolic genes, including those involved with protein (i.e., vitellogenin) synthesis, and modulators of molecular signaling pathways (e.g., Protein Kinase A) [21]. Exposure to nicotine, a related, but natural chemical resulted in an up-regulation of metabolic genes [22], suggesting an energetic cost for their detoxification. Complimentary anabolic and catabolic physiological processes, operated by insulin and octopamine signaling, respectively, regulate energetic and nutritional homeostasis in insects [23]. Release of neurotransmitters and neuromodulators (e.g., octopamine and insulin-like peptides (ILPs)) from brain neurosecretory cells may subsequently trigger a release of additional neruopeptides from the corpora cardiaca (CC), including adipokinetic hormones (AKHs), which have a primary function of regulating insect metabolism [24]. These and ILPs communicate with fat body cells, which mobilize necessary energetic substrates (e.g., lipids and glycogen). Energetic balance is reestablished after perturbation via several energetic-sensing, key regulatory switches of insect energetic balance (e.g., AMPK, c-AMP, PKA) that modulate the cellular response to stimuli via phosphorylation of cellular transcription factors [25] (e.g., FOXO proteins). These molecular signaling pathways are regulated both by axonic connections to the CNS and humoral communication via hormones. Some of these substrates/molecular signaling pathways are also involved with regulating the behavioral and physiological ontogeny of nurse bees as they transition to foragers [26,27,28,29,30].

Nurse bees, young adult workers that remain in-hive for 2–3 weeks prior to the onset of foraging, perform a number of important tasks during their development, including feeding young larvae and the queen, processing foods, building and maintaining beeswax comb, and guarding the hive [31]. Nurse bee developmental ontogeny is regulated by feedback between key substrates vitellogenin (vg) and juvenile hormone (JH); this regulatory module communicates with molecular signaling pathways (B-oxidation of lipids, octopamine, insulin) that regulate the physiological changes associated with the nurse–forager transition [26,27,28,29,30]. The intrinsic mode of action of neurotoxic neonicotinoids on neuronal regulated processes that maintain energetic and nutritional balance, together with interactions between these pathways and those underlying the stress response [32], make it particularly hard to distinguish between the direct effects of tested compounds on honey bee physiology, and the indirect effects of compounds by triggering a chronic stress response in exposed honey bees. Moreover, the involvement of many of these same molecular pathways in nurse bee development make understanding the effects of neonicotinoids on important markers of nurse honey bee nutrient and energetic physiology of high importance.

The goal of this study was to try to detangle the effects of the stress response from neonicotinoid exposure and their direct impact on neuronal pathways underlying nutrient and energetic balance of exposed honey bees. To this end, groups of nurse bees were orally exposed to either low or high doses of two neonicotinoid pesticides, clothianidin and imidacloprid, for two weeks. Measurements taken include food consumption, whole-body protein, lipid, and glucose and glycogen content of individual honey bees, and their respiration (metabolic rate). These markers are relevant to nurse bee behavioral ontogeny and thus to colony health and endurance.

## 2. Materials and Methods

### 2.1. Honey Bees and Experimental Conditions

In mid-April, commercial honey bee packages (Wilbanks Apiaries, Claxton, GA, USA), which contained a single fertile queen and approximately 2000 adult workers, were established in an apiary located on the grounds of the Beltsville Agricultural Research Center, Beltsville, MD. Honey bee colonies were housed in hives consisting of brand new 10-frame Langstroth boxes. Colony workers foraged naturally for local pollen and nectar, and the growth, overall health, and nutrient stores of colonies were recorded bi-weekly. In early July, fifty adult workers were collected from brood frames of each colony into separate 50 mL tubes, and then stored at −80 °C until processed for molecular diagnosis of natural microbial pathogen loads (USDA-ARS Honey bee Disease Diagnostics Laboratory, Beltsville, MD, USA). After receiving results, five colonies having similarly low loads of common honey bee pathogens (*Nosema* spp., deformed wing virus, etc.) were selected as sources of bees used in laboratory experiments.

For collection of same-aged honey bee cohorts for laboratory studies, a frame having large numbers of capped brood cells was retrieved from each of the selected field colonies, and kept separately in closed, screened wooden frame boxes placed in a dark incubator with temperature set at 33 °C and ~75% relative humidity (RH). Twenty-four to thirty-six hours later, hundreds of worker bees emerged from their cells. Hundreds of emergent adults were combined into a large tub, gently mixed, and collected into 25 groups of fifty bees each. Five cages housing 50 bees each were assigned to each of the five treatments (see Section 2.2). Fifteen additional newly emerged bees were also collected into a 15 mL tube and then stored at −80 °C until honey bee samples were used for measurements of baseline values for nutrient and energetic bioassays (see Section 2.3). Prior to placing in cages, three workers for each cage were marked on the dorsal thorax with a different color medium point uni Posca pen (Mitsubishi Pencil Co., LTD, Tokyo, Japan). These bees were followed over two weeks, and all measurements made were for these individuals. If a marked honey bee died in a cage during the experiment, a new resident honey bee was selected, marked and used in all subsequent measurements.

Honey bees were housed in experimental cages constructed from 0.5 mm wire mesh sheeting formed into a cylinder (17 cm height × 8.5 cm diameter). Wide-mouth Masson jar lids were used as the top and floor of each cage. Tops and bottoms were secured to the cage by inserting narrow-gauge metal wire through small holes drilled into opposite sides of the walls of each lid and the sides of the cage, and bent 90°. As a perch for bees, a 7 cm × 7 cm block of clean plastic comb was secured with wire mesh to the wall of each cage. Two holes (~2.5 cm) were drilled into the top lid, each for holding a 20 mL inverted glass scintillation vial (Wheaton). Cages were housed in an incubator as described above.

### 2.2. Experimental Foods and Pesticide Treatments

Each cage was provided with 2 vials, each having 20 mL of either 2 M (~56% *w*/*w*) sucrose (Sigma-Aldrich, St. Louis, MO, USA) (sugar) solution (hereafter, sugar syrup) or water; bees accessed sugar syrup and water through three small holes drilled into the lid of the inverted scintillation vials. Bees were also provided a ~2.5 g portion of lab-made pollen patty in a small plastic weigh boat placed at the cage bottom. Pollen patties (hereafter, PP) were made from BeePro^®^ pollen supplement augmented with fresh honey bee-collected multi-floral pollen, and sugar (Supplementary Materials, Table S1). After mixing ingredients together, the resultant dough was rolled out to form a sheet ~1 cm thick, then wrapped in wax paper and plastic wrap, and frozen at −20 °C until use. When ready, 1.5 cm diameter portions (~2.5 g) were cored from the dough using a hollow cylindrical metal punch.

Pesticides imidacloprid and clothianidin (Pestanal, Sigma-Aldrich, Darmstadt, Germany), each at 5 and 50 ppb (μg/kg) (treatments “IMLO” and “IMHI”, and “CLLO” and “CLHI”, respectively) were administered orally in both pollen patties and in sugar syrup. Selected doses are similar to field relevant concentrations of neonicotinoids determined in honey bee collected pollen and nectar [6,7,9]. The solubility of both neonicotinoids in water (imidacloprid: 0.51 g/L; clothianidin: 0.33 g/L) made it necessary to pre-dissolve imidacloprid and clothianidin each in a small volume of acetone before mixing with water to make a 1 mM stock solution for each pesticide. An appropriate amount of this stock solution was added to sugar syrup (*v*/*v*) and water used to make pollen patties (*w*/*w*). A “Control” treatment sugar syrup and pollen patty (each having no pesticides) were also formed with the addition of the mean volume of acetone added to either the PP or sugar syrup of the other treatments (<1 mL). Duplicate samples of each food type and treatment, including controls, were sent to the AMS USDA-ARS laboratory in North Carolina for analysis of their pesticide residue content using GC-MS/MS.

All foods and water were provided ad libitum, and consumption of each was recorded weekly for two weeks. Non-consumed foods were replaced each week regardless of consumption, and no microbial contamination was observed in foods at any time. Food consumption was calculated on a dry weight basis, and water consumption on a volume basis. Five cages containing no bees, but having vials containing both sugar syrup and water were also placed in the incubator alongside cages with bees, and their volumes monitored to determine amounts lost to evaporation. When applicable, these values were subtracted from values measured from vials in cages housing honey bees. The amount of sugar consumed (in mg) was calculated by multiplying the volume of sugar syrup consumed by 1.26 g/mL, the density of a 2 M sucrose solution. For quantifying PP consumption, fifteen 2.5 g portions of each PP were weighed, dried for 10 days, and then reweighed to calculate average evaporative weight loss over this time. Pollen patty dry-weight data were plotted against their wet weights, and the linear regression equation used to estimate the dry weights of pollen patty portions initially provided to bees. Pollen patty remains were placed with their weigh boats in a 50 °C drying oven for ten days. The dry weights of PP remain were then subtracted from their estimated initial dry weights to calculate the amount of dried PP consumed by bees.

Honey bee mortality was recorded, and any dead bees removed and/or remarked, on Days 4, 7, 10, and 14, over the course of the experiment. Numbers of dead bees were summed to give the total mortality after two weeks. Total mortality was included as a co-variate in both a MANCOVA testing treatment effects on total food consumption, and separate ANCOVA tests of treatment effects on total consumption of PP and sugar syrup. Student’s post hoc tests were run to determine between-treatment differences. Similar calculations were made to determine the amount of clothianidin or imidacloprid consumed by bees in each of the treatments by multiplying total food consumption values by the average amount of pesticide residues determined from GC-MS/MS analyses of food samples (above).

### 2.3. Nutrient and Energetic Bioassays

A spectrophotometric method [33] for closely approximating the content of soluble protein, lipids, simple carbohydrates (e.g., glucose), and glycogen from individual insects was adapted for use with honey bees. The fifteen newly emerged honey bees stored at −80 °C and two-week-old marked individuals from cages were each individually weighed (to 0.01 mg) on a Metler Toledo (model AL 104) balance. Then, each honey bee was placed in a separate 2 mL screw cap microcentrifuge tube (FastPrep, MP Biomedicals, Santa Ana, CA, USA) with approximately 0.2 mL (650 mg) of 2 mm diameter zirconium beads and 1.5 mL of phosphate buffered saline (PBS, pH 7.2). Samples were homogenized using a Precellys 24 tissue homogenizer, set at 6500 Hz for 20 s. Afterwards, tubes were centrifuged at 3000 rpm at 4 °C for 5 min to remove large pieces of tissue from the supernatant.

The Bradford assay [34] was used to determine soluble protein content in honey bee tissue homogenates. First, 50 μL of the supernatant was carefully removed to a new tube, diluted 10X with PBS, and vortexed well. From this tube, 5 μL was pipetted into separate duplicate wells of a flat-bottomed, clear 96-well polypropelene microplate (Fisher Scientific, Hampton, NH, USA). Next 245 μL of 1X Coomessie dye (Sigma Aldrich, St. Louis, MO, USA) was added to each well and homogenized at 300 rpm for 15 min on a Fisher Scientific vortex fit with a microplate attachment. Eight bovine serum albumin (BSA) (Sigma Aldrich, USA) standards ranging 0–2.0 mg/mL were treated similarly as samples. Sample and standard absorbance was read at a wavelength set at 595 nm using a BioTek H1N multimodal microplate reader.

The vanillin assay [35] was used to determine lipid content in honey bee tissue homogenates. First, 100 μL of the supernatant was pipetted into a new 2 mL microcentrifuge tube and then diluted 1:3 in PBS. Next, 180 μL of the diluted supernatant was mixed with 20 μL of 20% sodium sulfate solution (Sigma-Aldrich, USA) and 1.5 mL of 1:2 chlorform:methanol to solubilize the carbohydrates and lipids, respectively. The tube was vortexed vigorously for 1 min then centrifuged at 3000 rpm at 4 °C for 15 min. For the vanillin assay, 100 μL of each sample and eight standard triolein (Sigma Aldrich, USA) solutions in 1:2 chlorform:methanol, ranging in concentration from 0 to 1.0 mg/mL, were pipetted into duplicate wells of a flat-bottomed, clear 96-well quartz microplate (Hellma Analytics, Müllheim, Germany), and placed on a 90 °C heat block until complete solvent evaporation. Afterwards, 20 μL of 98% sulfuric acid was added to each well, and the microplate sealed with an adhesive aluminum sheet. The microplate was incubated in a very shallow 90 °C water bath for 2 min, and then cooled on ice. Next, 180 μL of freshly prepared vanillin reagent (1.2 g/L in 68% phosphoric acid; Sigma Aldrich, USA) was added to each well, and contents homogenized using a vortex fitted with a microplate adapter at room temperature for 15 min. Finally, the microplate was read at an absorbance wavelength set at 525 nm.

The anthrone assay [36] was used to determine the content of both glucose and glycogen in tissue homogenates. First, for the glucose assay, 20 μL of the 1:3 diluted supernatant (above) was pipetted into separate wells of a quartz microplate, and then 230 μL of freshly prepared anthrone reagent (1.42 g/L in 70% sulfuric acid; Sigma Aldrich, USA) was added to each well. Eight glucose (Sigma Aldrich, USA) standards ranging 0–1.0 mg/mL were treated the same as samples. The microplate was then carefully covered with an adhesive aluminum sheet and incubated at room temperature for 15 min. Next, the microplate was placed in a very shallow 90 °C water bath for 15 min, after which the microplate was read at an absorbance wavelength set at 625 nm.

For determining glycogen content in samples, the remaining supernatant (above) was removed from microcentrifuge tubes, leaving behind a pellet containing precipitated glycogen bound to sodium sulfate. The pellet was washed by two rounds of vigorous vortexing in 500 μL of fresh 80% methanol followed by centrifugation at 13,000 rpm at 4 °C for 15 min. After the final wash, methanol was removed with a micropipette, and then 1 mL of anthrone reagent added to each tube. The samples were homogenized by vortexing for 1 min, and then placed in a 90 °C water bath for 15 min. After cooling on ice, each sample was passed through separate low-protein binding membranes (polyvinylidene fluoride; d = 0.45 μm, Millipore, USA) fit to 3 mL volume syringes. After filtration, 250 μL of each sample was pipetted into separate wells of a 96-well quartz microplate. Eight glycogen (from oyster, Sigma Aldrich, USA) standards were prepared and treated the same as the glucose standards (above). The microplate was read at an absorbance wavelength set at 625 nm.

To determine the quantity (in mg) of each nutrient and energetic substrate in honey bee tissues, concentrations of each substrate, in mg/mL calculated from their respective standard curves, were multiplied by dilution factors, and then by 1.5 mL to obtain quantities of substrates from the whole bee homogenate. Data of each substrate were plotted against body weight to determine whether data scaled significantly with body weight. If so, data analyses included these weight-adjusted values. Data were visualized and tested against requirements of parametric statistics, and were transformed where necessary to meet these requirements. Non-parametric statistical methods were used for analyses of data non-normalized using common data transformations.

### 2.4. Honey Bee Respirometry

Carbon dioxide (CO_2_) emission was measured using closed-system flow-through respirometry [37]. Baseline respiration of marked bees (see above) was measured within 24–36 h post-emergence from brood cells. The weight of each bee was recorded, and then each placed into a separate airtight 7 cm by 2 cm cylindrical glass insect respirometry chamber (Sable Systems International, Las Vegas, NV, USA). Bees were able to walk, but not fly, freely around inside the chamber. Chambers were connected with Bevaline tubing (~0.5 cm outer diameter) to an 8-channel multiplexer (RM-8 Multiplexer; Sable Systems). The sequence in which individual bees from each treatment were assayed for CO_2_ emission was haphazardly assigned to avoid any effect of time between being placed in respirometry chambers and when their CO_2_ emission was measured. An empty, but otherwise identical respirometry chamber was used to measure baseline CO_2_ values of incurrent air. Respirometry chambers were housed in temperature-controlled cabinet (PELT-5; Sable Systems) set at 33 °C.

A flow rate of 150 mL/min of dry, CO_2_-free compressed air was maintained with a mass-flow controller (Tylan model FC-2900V; Sierra Instruments, Monterey CA, USA; 500 sccm, calibrated for air), connected to an MFC-2 mass flow valve controller (Sable Systems). Prior to measuring CO_2_ emission, honey bees were kept in chambers connected to the airstream for 10 min. Carbon dioxide emission was measured for 10-min intervals, using a CA-10A duel-wavelength infrared optical bench CO_2_ analyzer (Sable Systems). The analyzer was calibrated routinely with dry, pure N_2_ and 0.1% CO_2_ balanced with N_2_ (Airgas). Respirometric data, as the volume of CO_2_ released per time interval, were acquired using standard equations using an incurrent flow rate [35]. Data were logged using ExpeData software (version 1.3.0), receiving digital signals from an analogue-to-digital data converter (UI2, Sable Systems). Water vapor was added into the incurrent (dry) airstream by passing air through a 500 mL Erlenmeyer flask with a rubber stopper having two holes punched in the top for allowing an incurrent and an excurrent flow from the flask. Water vapor was scrubbed from excurrent airstream using magnesium perchlorate (Sigma Aldrich, USA) prior to measuring CO_2_.

Respirometry was measured again for the same marked bees (see above) on Day 14 of the experiment. For taking measurements, marked bees were removed from the cages by cooling caged bees in a refrigerator for 5–10 min to reduce honey bee activity. The measured metabolic rate of individual bees is recorded as the milliliter of CO_2_ emitted per minute (VCO_2_). Log-log regression analyses of body mass and VCO_2_ were conducted to determine body weight–respiration relationship. If significant, mass-specific metabolic rates were calculated, and these values used in analyses with treatment as an additional factor. Student’s post hoc tests were used to separate effects across treatments.

## 3. Results

### 3.1. Food and Pesticide Consumption

**Figure 1:**
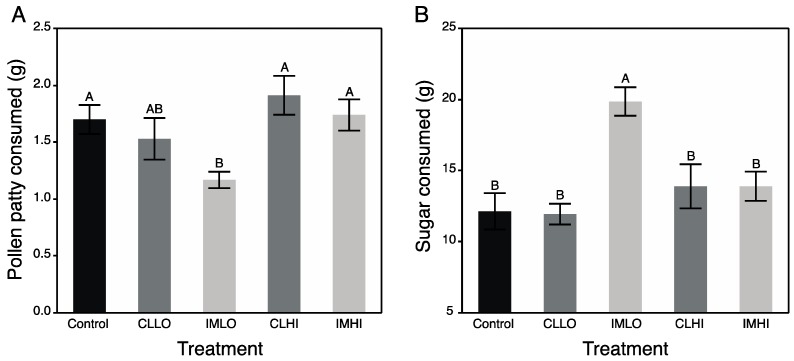
Mean (±s.e.m) total pollen patty (**A**) and sucrose (**B**) from sugar syrup consumed by caged honey bees over two weeks. Black bar = Control treatment; Dark grey bars = Clothianidin Low (CLLO) and High (CLHI) dose treatments; Light grey bars = Imidacloprid Low (IMLO) and High (IMHI) dose treatments. Letters above bars represent significant differences across treatments from Student’s post hoc tests.

The total number of dead bees (log_10_-transformed) was not significantly different across treatments (F_4,20_ = 0.78; *p* = 0.551) (Figure S1). Before the two-week experimental period elapsed, eight marked bees perished, representing a loss of at least one bee from each treatment. Results from MANCOVA revealed that total food consumption was significantly different across treatments, and significantly affected by the number of dead bees. In addition, from separate ANCOVAs, total PP and sugar syrup consumption was also significantly different across treatments, but the number of dead bees did not significantly affect PP or sugar consumption (Table 1). Treatment “IMLO” bees consumed significantly less total food and PP, but more sugar syrup, compared to “Control” treatment bees and those exposed to high doses of both pesticides. Treatment “CLLO” bees consumed similar amounts of PP as “IMLO” treatment bees, but also similar amounts as bees from other treatments (Figure 1A). Bees exposed to a low dose of imidacloprid consumed significantly more sugar syrup than bees from all other treatments (Figure 1B).

Data of pesticide recovery from GC-MS/MS analyses of duplicate food samples are presented in Table S2. Duplicate values were averaged, and these values multiplied by the amount (g) of either food type consumed. Data for each food type were summed to obtain the total amount of pesticide consumed (Table S2). Pesticide consumption was significantly different across treatments (not including “Control”) in a Kruskal–Wallace test (χ^2^ = 16.2; df = 3; *p* = 0.001). Results from a Wilcoxon post hoc test showed separation between low and high dose treatments, with bees from either low or high dose treatments consuming similar amounts of their respective pesticides. The intake of clothianidin and imidacloprid in high-dose treatments averaged ~30% of the LD_50_ for these compounds, and ~10 and ~20 times below the LD_50_ value, respectively, for low-dose clothianidin and imidacloprid treatments [36].

### 3.2. Energetic and Macronutrient Bioassays

**Figure 2:**
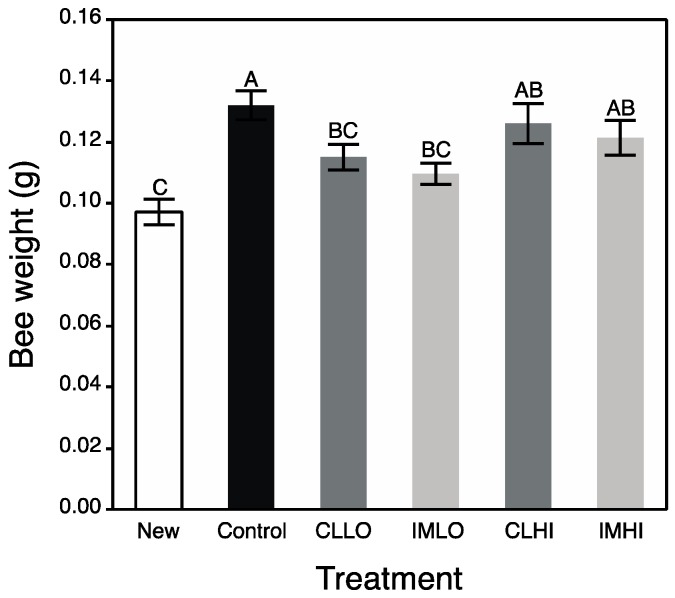
Mean (±s.e.m) weight of individual newly emerged honey bees and those from pesticide treatment groups after two weeks. White bar = Newly Emerged Treatment; Black bar = Control treatment; Dark grey bars = Clothianidin Low (CLLO) and High (CLHI) dose treatments; Light grey bars = Imidacloprid Low (IMLO) and High (IMHI) dose treatments. Letters above bars represent significant differences across treatments from Student’s post hoc tests.

Exposure to low doses of both neonicotinoids resulted in reduced bee body weight (*F*_5,84_ = 7.23; *p* < 0.001). Results from Student’s post hoc tests revealed that “CLLO” and “IMLO” treatment bees were significantly lighter in weight than “Control” treatment bees, and were intermediate in body weight between “Control” treatment bees and bees from other pesticide treatments (Figure 2).

**Figure 3:**
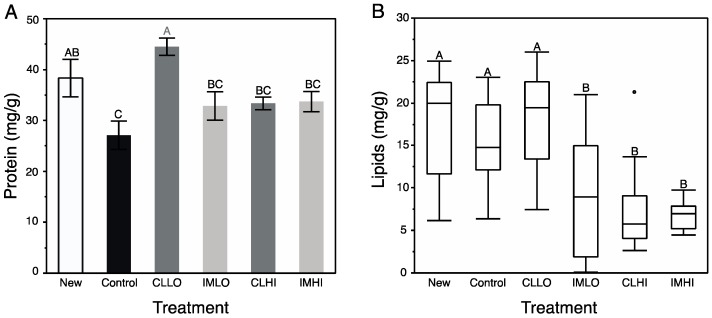
Mean (±s.e.m) relative quantity (mg/g) of soluble proteins (**A**) and median (±quartiles) lipids (**B**), from spectrophotometric assays of homogenized tissues of individual newly emerged honey bees, and those from pesticide treatment groups after two weeks. (**A**) White bar = Newly emerged bees; Black bar = Control treatment; Dark grey bars = Clothianidin Low (CLLO) and High (CLHI) dose treatments; Light grey bars = Imidacloprid Low (IMLO) and High (IMHI) dose treatments. Single point in (**B**) represents outlying data point. Letters above bars represent significant differences across treatments from Student’s (**A**) or multiple comparison Wilcoxon (**B**) post hoc tests.

Soluble protein content was significantly different across treatments (Table 2), and increased with larger bee weights (*r^2^* = 0.08; *p* = 0.015). Results from a post hoc test indicated that treatment “CLLO” bees contained similarly high levels of protein as newly emerged bees, and both groups contained more soluble protein than “Control” treatment bees. Bees from all other treatments contained protein intermediate to “Control” treatment and newly emerged bees (Figure 3A).

Honey bee lipid content was significantly different across treatments (Table 2) and lipid content increased positively with larger bee weights (*r^2^* = 0.06; *p* = 0.047). A post hoc analysis revealed newly emerged, “Control”, and “CLLO” treatment bees contained similarly larger amounts of lipids compared to bees from the other treatments (Figure 3B).

**Figure 4:**
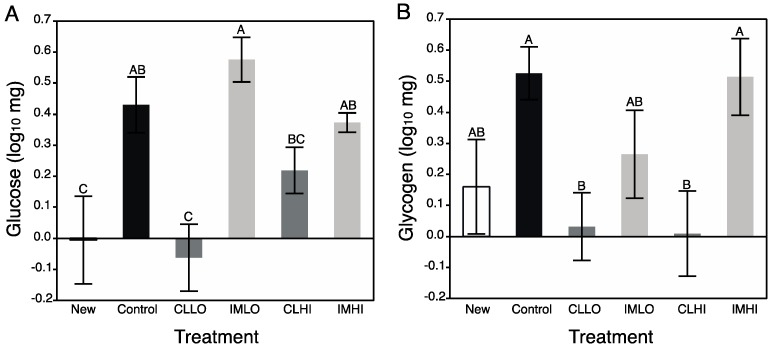
Mean (±s.e.m) log_10_-transformed quantity of glucose (**A**) and glycogen (**B**), measured by spectrophotometric assays of homogenized tissues of individual newly emerged honey bees, and those from pesticide treatment groups after two weeks. White bar = Newly emerged bees; Black bar = Control treatment; Dark grey bars = Clothianidin Low (CLLO) and High (CLHI) dose treatments; Light grey bars = Imidacloprid Low (IMLO) and High (IMHI) dose treatments. Letters above bars represent significant differences across treatments from Student’s post hoc tests.

Data of both honey bee glucose and glycogen content were log_10_-transformed prior to running separate ANOVAs. Honey bee glucose and glycogen content were both significantly different across treatments (Table 2), and did not significantly correlate with bee body weight (*r^2^* = 0.02; *p* = 0.228 and *r^2^* = 0.01; *p* = 0.553, respectively). Post hoc analyses revealed newly emerged and treatment “CLLO” bees contained significantly less glucose than bees from all other treatment groups (Figure 4A). For glycogen content, post hoc analysis revealed “Control” treatment bees contained significantly more glycogen than newly emerged bees, and bees from “CLLO” and “CLHI” treatment groups. Imidacloprid exposed bees had glycogen levels intermediate between, and not significantly different from “Control” treatment and newly emerged bees (Figure 4B).

### 3.3. Respirometry

**Figure 5:**
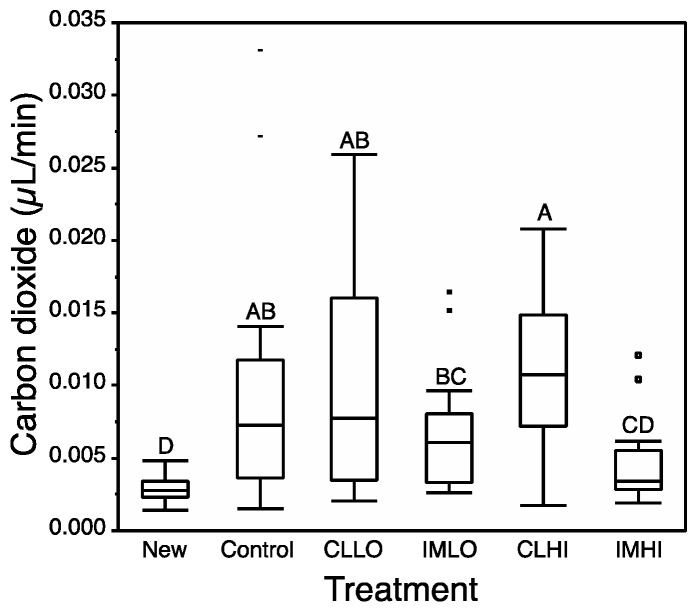
Median (±quartiles) volume of carbon dioxide (μL/min) emitted over 10 min by individual newly emerged honey bees, and by honey bees after two weeks exposure to two neonicotinoids. Treatment groups = Clothianidin Low (CLLO) and High (CLHI) dose treatments; Imidacloprid Low (IMLO) and High (IMHI) dose treatments. Letters above bars represent significant differences across treatments from multiple comparison Wilcoxon post hoc tests.

Respiration rate (VCO_2_) did not significantly correlate with log_10_-transformed bee weights, including separate tests for newly emerged and two-week-old bees (data not shown). Thus, bee weight was not included in a co-factor in non-parametric tests of treatment effects on honey bee respiration. Data were first tested against bee age, and then, omitting newly emerged bees, data were tested against treatment (Table 3). Bees from all treatments, except “IMHI” treatment bees, had significantly higher respiration rates than newly emerged bees; treatment “IMHI” bees had only slightly higher respiration rates than that of newly emerged bees (Figure 5). 

## 4. Discussion

Results from this laboratory study suggest that imidacloprid and clothianidin impacted energetic/nutrient homeostasis of exposed honey bees, including food consumption, in a complex compound and dose-dependent manner. These results may be possible from confounding (vis-à-vis “Control”) effects of the neonicotinoid compounds on catabolic and anabolic physiological processes of honey bees. Signaling pathways involving octopamine and AKH (adipokinetic hormone), and insulin/insulin like peptides (ILPs)-insulin growth factor (IGF) (IIS-IGF-1 signaling pathway), regulate insect energetic/nutrient homeostasis, growth, and can promote catabolic and anabolic physiological processes, respectively. Feedback loops and allosteric interactions reestablish nutrient and energetic balance after perturbation via a number of molecules including nutrient and energetic-sensing, key regulatory switches of insect energetic balance (e.g., nutrient levels, insulin, AMPK, c-AMP, PKA). Some of these modulate the cellular response to stimuli via phosphorylation of cellular transcription factors [24]. Many of these same pathways are also included in the honey bee stress response [32,38], and are also involved, together with the vg-JH regulatory module, in regulating the behavioral and physiological development of young adult honey bees [26,27,29,39,40,41,42,43,44]. Below, results are discussed in terms of how these pathways may have been affected by neonicotinoid exposure, and how compound and dose-dependent differences in behavior and physiology might have arisen. In addition, results are discussed in terms of possible secondary effects from the stress response and interactions with the vg-JH regulatory module that may have ramifications to the developmental ontogeny of young adult honey bees.

### 4.1. Compound and Dose-Dependent Responses

The most notable compound-associated results include effects of clothianidin exposure on carbohydrate metabolism, and effects of imidacloprid on respiration. Compound effects could arise from a number of factors, including an intrinsic ability of compounds to affect nAChRs activity [45,46], slight differences in three-dimensional structure of receptors themselves may also influence the affinity of neonicotinoids for binding to these receptors [5,47], their accessibility to receptors (i.e., their hydrophobicity) [48], and the detection sensitivity and metabolism of honey bee detoxification system to these compounds [49]. Although imidacloprid and clothianidin are chemically similar nitroguanidine neonicotinoids, they differ in their three-dimensional configurations [4], which may influence their potency and effectiveness, and the types of receptors to which they may irreversibly bind [46]. Clothianidin is a stronger agonist of nAChRs of the CNS than imidacloprid, binding to multiple types of these receptors [46,47], but imidacloprid is less hydrophobic than clothianidin, possibly allowing this compound to access receptors that the latter compound cannot [48] (e.g., hemocoel located receptors). Greater hydrophobicity can enhance transport of compounds to target sites [3]. Compound effects may also be related to whether humoral and/or CNS controlled aspects of physiology were impacted (i.e., neurohormones released from other sites (e.g., gut or fat body)), which may be related in part to their physical properties (hydrophobicity).

The most notable dose-dependent results include low-dose imidacloprid affecting feeding behavior, high-dose imidacloprid affecting respirometry, and, for both compounds, low-dose affecting body size. Dose-dependent differences might arise from the extent to which the compounds were perceived and detoxified [49,50] and/or whether all compounds were detoxified in the gut before becoming absorbed into the hemocoel. Dose-dependent differences may also arise from how levels of affected compounds influence secondary molecular pathways, specifically those related to the stress response. For example, release of octopamine can act on downstream responses in a dose-dependent fashion, with high and low doses stimulating and inhibiting downstream responses, respectively (e.g., [51]).

### 4.2. Food Consumption

Food is necessary for promoting maintenance and growth of living systems. Young adult honey bees consume copious amount of pollen, which is rich in protein and lipids, for 7–10 days post emergence ([52], and authors’ observation), in part to supply the hypopharyngeal glands (HPs) that produce nutrient dense secretions (royal jelly) fed to queens and young larvae [53], and also to further their adult development. After this time, protein consumption by nurse bees drops precipitously, and carbohydrate consumption sharply rises ([52], and author’s unpublished data). This switch in nutrient preferences is likely regulated by the molecular signaling cascade that modulates the behavioral ontogeny of nurse bees (i.e., vg and JH regulatory module).

In the present study, treatment “IMLO” bees showed an opposite pattern in nutrient preferences, consuming much less PP and much more sugar syrup than bees on most other treatments (“CLLO” treatment bees consumed similar amounts of PP). Altered protein nutrition may also be seen in field bumble bee colonies, which reduced pollen foraging after sub lethal exposure to imidacloprid [54]. Imidacloprid exposure can also reduce the size of HP gland acini [55], as can poor nutrition (pollen restriction) [52]. Consumption of much less protein/lipids in PP may have resulted in the lower body weights of “IMLO” treatment bees, and more water-soluble proteins, rather than tissue-bound proteins measured in “CLLO” treatment bees, could help explain why the latter bees had reduced body weights. These differences may also arise from affected molecular pathways that regulate insect growth. Because both “CLLO” and “IMLO” treatment bees were significantly lower weight than those from other treatments, this suggests aspects of the Insulin/Insulin Growth Factor (IIS) signaling pathways may have been altered from low dose exposure, perhaps from inhibitory effects on this pathway from low levels of octopamine (above), which may have been higher in high-dose neonicotinoid treatments. Insulin signaling can affect carbohydrate metabolism. Indeed, some “IMLO” treatment bees upon freezing regurgitated crop contents, which were frozen and attached to the bee’s mouthparts (authors’ observation). Additionally, a few observations were made of sugar syrup being stored by bees in cells of plastic comb placed in cages. In other studies, both honey bees and bumble bees consumed more sugar water containing neonicotinoids [56,57] and honey bees from field colonies preferred sugar water resources with low levels of nicotine [58]. One explanation to the overconsumption of sugar water may be that imidacloprid is attaching to nicotinic acetylcholine receptors of gustatory cells, which when stimulated, release neuropeptides into hemolymph that then communicate with insulin producing brain neurosecretory cells (IPCs) to release ILPs. However, both honey bees and bumble bees were apparently unable to detect neonicotinoids in the sugar water with either olfactory or gustatory sensillia [56], suggesting the compounds may have impacted other pathways regulating satiety (e.g., gut and/or brain). In this study, overconsumption of sugar syrup by “IMLO” treatment bees suggests imidacloprid affected physiological processes associated with the IIS-IGF-1 signaling pathway. In *Drosophila*, down-regulation of insulin signaling can lead to fed flies feeding more, and on less nutritious foods [59].

### 4.3. Energetic and Macronutrient Bioassays

Generally, results from bioassays of nutrient and energetic substrates showed neonicotinoid exposure favored catabolic over anabolic physiological processes in young honey bees. Signaling pathways involving octopamine, AKH, and insulin regulate insect energetic/nutrient homeostasis, and can promote catabolic and anabolic physiological processes, respectively. The exception to the generality is treatment “CLLO” bees, which appeared to remain juvenile compared to newly emerged bees and bees from other treatments after two weeks of exposure. These results suggest that the included neonicotinoids differentially affected these physiological processes. For “CLLO” bees, protein content remained at levels similar to newly emerged bees. Typically, body protein decreases over nurse bee behavioral ontogeny ([60] and author’s unpublished data), which is correlated with both reduced protein consumption and production of vg [61], a water-soluble hexameric storage protein. Vitellogenin plays unique role in honey bee development, and interacts inversely with JH titers to regulate the temporal transition of nurse bees to foragers [40] (i.e., as vg levels drop, JH levels rise, as reviewed in [41]). Neonicotinoid exposure has been shown to affect both vg levels and protein metabolism of honey bees. For example, bodies of nurse bees exposed to imidacloprid had reduced vg levels compared to similarly-aged unexposed counterparts [62], and proteolytic activity and protein content appears dose-dependent in imidacloprid-exposed bees [63]. Changes in vg synthesis may alter JH levels and affect downstream signaling pathways [41]. Very high measured protein levels of “CLLO” treatment bees suggest that vg production is not down-regulated, and thus JH levels may be expected to be high. Higher protein levels in “CLLO” treatment bees may also be associated with an uptick in levels of stress response proteins (e.g., heat shock proteins, catalase), which are higher in neonicotinoid exposed honey bees [21].

Lipid content of exposed bees compared to that of “Control” treatment bees was significantly reduced, except for treatment “CLLO” bees, which had lipid levels similar to those of newly emerged bees. Through a physiological process (involving JH and insulin) of stable lipid loss, lipid content decreases in nurse bees as they transition to foragers [30,43]. Lipolysis can also mobilize lipids in response to energetic demands, including those associated with the stress response via release of octopamine from brain neurosecretory cells [38]. Additionally, systemically administered nicotine induced lipolysis via activation of the classical adrenergic, stress-induced pathway (e.g., via octopamine, IIS signaling), and also by directly activating a nicotinic cholinergic receptor located in adipose tissue [64], which is replete with cholinergic receptors [25]. Thus, reduced lipid content in treatment “IMLO”, “IMHI”, and “CLHI” bees may be from either or both mechanisms. Accelerated lipid loss is another factor that may result in precocious foraging of nurse honey bees [30]. Sugars consumed in excess of demands may be stored as lipids, but for at least treatment “IMLO” bees, which collected relatively large volumes of sugar syrup, this was not observed. Although lipid levels of treatment “CLLO” bees were not significantly different than “Control” treatment bees, it was not reduced similarly to those on other pesticide treatments. This discrepancy may be related to the effect of JH on lipolysis; the vg-JH regulatory module appears compromised in treatment “CLLO” treatment bees, thus also affecting lipid levels.

Carbohydrate metabolism was severely impacted by clothianidin exposure; treatment “CLLO” and “CLHI” treatment bees showed reduced glucose and glycogen levels. AKH and insulin-signaling molecular pathways regulate carbohydrate metabolism for energy production, and these pathways can interact with the TOR signaling and FOXO pathways to affect energetic balance. In contrast to lipids, sugars consumed in excess may be stored internally as glycogen. For “IMLO” treatment bees, consumption of large volumes of sugar syrup slightly increased glycogen stores. Glycogen is an important energy storage molecule in animals, and is often used in instances that require a ready source of metabolic energy, such as during honey bee flight [65], or heat production during winter [66]. Glycogen synthesis proceeds by glucose polymerization via glycogen synthase, which is regulated by allosteric effectors, such as glucose-6-phosphate, by phosphorylation reactions with neuronal peptides (e.g., glycogen synthase kinase (GSK-3), AMPK, protein kinase A (PKA), and indirectly via insulin inhibition of phosphorylating substrates. Protein kinase A levels were differently affected by clothianidin and imidacloprid exposure, with PKA and *creb* transcript being down regulated in clothianidin-exposed, but not imidacloprid-exposed bees [21]. This suggests a mechanism to understand the altered carbohydrate metabolism observed in, especially, “CLLO” treatment bees. Somewhat recovered glycogen storage levels in “CLHI” treatment bees may be a result of an alternative pathway resulting in glycogen storage. This may be a result of the stress response with octopamine and AKH helping to increase storage of glycogen.

Little is certain of how glycogen levels change during nurse bee development, but glycogen synthesis declines in flight muscles of foraging honey bees as they age [67]. Given that newly emerged bees contained very little glycogen and young foragers contain abundant glycogen, it is likely that during nurse bee ontogeny glycogen levels rise. It is not completely clear what neonicotinoid exposure and its effect to honey bee glycogen levels might have at the colony level; newly foraging bees with low levels of glycogen may not replenish these stores, thus potentially reducing their longevity or suitability as foragers. The very low levels of glucose measured for “CLLO” and “CLHI” treatment bees could explain their reduced glycogen content. However, for these bees, low sugar content did not reflect low consumption of sugar syrup, and might be a symptom of a maintaining a somewhat high metabolic rate, which would use up ingested sugars.

### 4.4. Respiration

Imidacloprid exposure had the greatest impact on honey bee respiration. By Day 14, only “IMHI” treatment bees showed a depressed respiration rate. As nurse bees become active foragers, their cellular respiration rate is likely to increase [68]. Carbon dioxide emission rates measured in our study for newly emerged bees correspond to values measured for resting honey bees [69], and also correspond to rates measured for high-dose imidacloprid exposed bees. Hatjina et al., [55] observed a low dose of imidacloprid significantly reduced the abdominal ventilation rate of exposed honey bees, which can correlate with reduced respiration [68]. Reduced JH levels can reduce metabolic rate [42]. Low respiration rate might suggest a slow uptake of glucose into cells for glycolytic function. Reduced respiration suggests cells may lack access to glucose via disruption of insulin signaling or other molecular signals that regulate ATP use and production (e.g., c-AMP and AMPK). Despite low cellular respiration, “IMHI” treatment bees consumed similar amounts of sugar syrup as “Control” treatment bees, and had low glucose body content; it appears sugar consumed by “IMHI” treatment bees but not hydrolyzed in glycolysis, were packaged and stored as glycogen, levels of which were not reduced as seen in bees from the other treatments.

## 5. Conclusions

Taken together, the data presented here show a body-wide, multi-faceted effect of neonicotinoid exposure on physiological markers of individual honey bees. Protein, lipids, and carbohydrate content of body tissues may change predictably throughout nurse honey bee behavioral and physiological development. Deviations in the relative levels of these markers may signal perturbations from stress experienced by individual honey bees. The overlap between molecular signaling pathways that regulate nurse bee development and the stress response make exposure to endocrine-disrupting neurotoxins, such as neonicotinoids, particularly hazardous to individual bee and thus colony health [26,27,38,39,42]. Neonicotinoid exposure may not be the single factor attributed to the decline of honey bee colony health, but rather a component of a number of inter-related stress-inducing factors (e.g., pesticide exposure, disease, and poor nutrition), resulting in precocious foraging, which is a significant factor that may lead to colony collapse [19,70,71].

## Figures and Tables

**Table 1:** Results from a multivariate statistical test of experimental Treatment effects on total amount of food consumed, and separate univariate tests on pollen patty and sucrose in sugar syrup consumed, by caged honey bees over two weeks.

Variable	DF	*F*	*p*
Total food	10, 36	5.99	<0.001
Treatment	8	6.99	<0.001
Dead bees *	2	4.04	0.035
Pollen patty	5, 19	3.83	0.014
Treatment	4	4.55	0.009
Dead bees *	1	2.54	0.128
Sugar syrup	5, 19	6.87	0.001
Treatment	4	7.65	0.001
Dead bees *	1	1.81	0.194

* log-transformed.

**Table 2:** Results from separate statistical tests of experimental treatments on energetic and nutrient substrate content of individual honey bees after two weeks.

Variable	DF	*F/X* ^2^	*p*
Protein	5.84	5.49	<0.001
Lipids ^†^	5.84	29.61	<0.001
Carbohydrate *	5.84	7.11	<0.001
Glycogen *	5.84	4.52	0.001

* log-transformed. ^†^ Wilcoxon non-parametric test.

**Table 3:** Results from statistical tests of pesticide treatments on honey bee respiration after two weeks.

Source	N	DF	*Χ* ^2^	*p*
Bee Age	141	1	46.02	<0.001
Treatment	73	4	10.70	0.030

## Supplementary Materials

The following are available online at http://www.mdpi.com/2075-4450/10/1/18/s1, Figure S1: Honey bee mortality, Table S1: Components and composition of pollen patties provided to caged honey bees, Table S2: Results from pesticide analyses (GS-MS/MS) of clothianidin and imidacloprid residues in pollen patties and sugar syrup provided to honey bees in experimental cages.
