# Supplementary material for: Compound and Dose-Dependent Effects of Two Neonicotinoid Pesticides on Honey Bee (Apis mellifera) Metabolic Physiology
Source: Insects. 2019 Jan 8;10(1):18. doi: 10.3390/insects10010018 (PMC6358842; doi:10.3390/insects10010018)
Supplement: Supplementary file 1 [file insects-10-00018-s001.zip › Neos Table S2.docx]

Table S2. Results from pesticide analyses (GS-MS/MS) of clothianidin and imidacloprid residues in pollen patties and sugar syrup provided to honey bees in experimental cages.

| Treatment | Food type | Sample | ng/g | Factor (mean ng/g) | Pesticide consumed (ng) |
| --- | --- | --- | --- | --- | --- |
|  |  |  |  |  |  |
|  | Pollen patty | 1 | N.D | 0 | 0 |
| Control |  | 2 | N.D |  |  |
|  | Sugar syrup | 1 | N.D | 0 | 0 |
|  |  | 2 | N.D |  |  |
|  | pollen patty | 1 | 8.2 | 4.6* | 7.0 ± 0.8 |
| Clothianidin 5 ppb |  | 2 | N.D |  |  |
|  | Sugar syrup | 1 | 1.2 | 0.9* | 10.8 ± 0.7 |
|  |  | 2 | N.D |  |  |
|  | Pollen patty | 1 | 39.4 | 41.9 | 80.1 ± 7.1 |
| Clothianidin 50 ppb |  | 2 | 44.4 |  |  |
|  | Sugar syrup | 1 | 28.4 | 34.5 | 483.4 ± 53.9 |
|  |  | 2 | 40.6 |  |  |
|  | Pollen patty | 1 | 5.5 | 6.2 | 7.3 ± 0.5 |
| Imidacloprid 5 ppb |  | 2 | 7.0 |  |  |
|  | Sugar syrup | 1 | N.D | 0.9* | 18.0 ± 0.9 |
|  |  | 2 | 1.4 |  |  |
| Imidacloprid 50 ppb | Pollen patty | 1 | 62.9 | 62.1 | 108.1 ± 8.5 |
|  |  | 2 | 61.3 |  |  |
|  | Sugar syrup | 1 | 37.8 | 36.7 | 514.2 ± 38.1 |
|  |  | 2 | 35.6 |  |  |
