# Supplementary material for: Compound and Dose-Dependent Effects of Two Neonicotinoid Pesticides on Honey Bee (Apis mellifera) Metabolic Physiology
Source: Insects. 2019 Jan 8;10(1):18. doi: 10.3390/insects10010018 (PMC6358842; doi:10.3390/insects10010018)
Supplement: Supplementary file 1 [file insects-10-00018-s001.zip › Neos mortality figure S1.pdf]

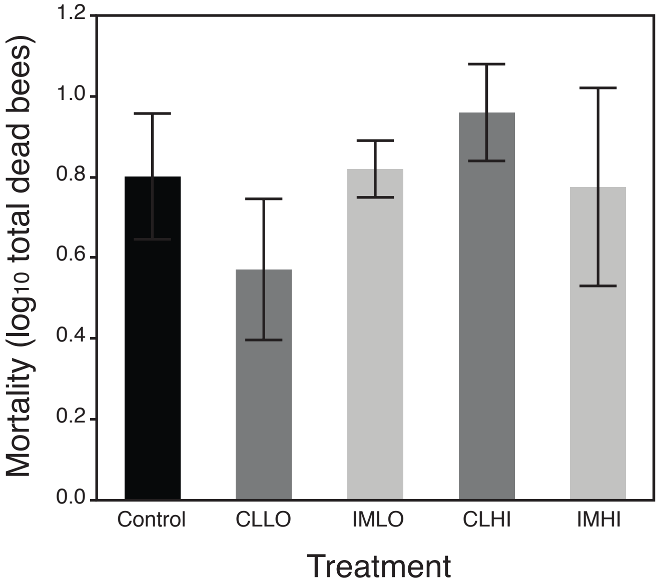

Figure S1. Mean (± s.e.m) $\log_{10}$-transformed number of dead honey bees in cages after two weeks of oral exposure to two neonicotinoids. Black bar=Control treatment; Dark grey bars=Clothianidin Low (CLLO) and High (CLHI) dose treatments; Light grey bars=Imidacloprid Low (IMLO) and High (IMHI) dose treatments.
