# Supplementary material for: Compound and Dose-Dependent Effects of Two Neonicotinoid Pesticides on Honey Bee (Apis mellifera) Metabolic Physiology
Source: Insects. 2019 Jan 8;10(1):18. doi: 10.3390/insects10010018 (PMC6358842; doi:10.3390/insects10010018)
Supplement: Supplementary file 1 [file insects-10-00018-s001.zip › neos Table S1.docx]

Table S1. Components and composition of pollen patties provided to caged honey bees over two weeks.

| Diet component | % Total (dw) | % Protein | % Carbohydrate |
| --- | --- | --- | --- |
| Sucrose | 47.4 | 0.0 | 100.0 |
| BeePro® | 45.6 | 48.5 | 0.0 |
| Pollen* | 8.0 | 40.0 | 10.0 |
|  |  |  |  |

* Nutrient content was taken from [30].
